# Neutrophil levels upon admission for the assessment of acute pulmonary embolism with intermediate- and high-risk: an indicator of thrombosis and inflammation

**DOI:** 10.1186/s12959-023-00471-w

**Published:** 2023-03-14

**Authors:** Rui Peng, Weihua Yin, Fang Wang, Xiangfeng Cong, Bin Lu, Lu Hua, Xi Chen

**Affiliations:** 1 Center of Clinical Laboratory, Beijing Friendship Hospital, Capital Medical University, Beijing, China; 2 Diagnostic Laboratory Service, State Key Laboratory of Cardiovascular Disease, National Center for Cardiovascular Diseases, Fuwai Hospital, Chinese Academy of Medical Sciences and Peking Union Medical College, Beijing, China; 3 Department of Radiology, State Key Laboratory of Cardiovascular Disease, National Center for Cardiovascular Diseases, Fuwai Hospital, Chinese Academy of Medical Sciences and Peking Union Medical College, Beijing, China; 4 Key Laboratory of Pulmonary Vascular Medicine & State Key Laboratory of Cardiovascular Disease, National Center for Cardiovascular Diseases, Fuwai Hospital, Chinese Academy of Medical Sciences and Peking Union Medical College, Beijing, China

**Keywords:** Neutrophils, Acute pulmonary embolism risk classification, Inflammation, Thrombosis

## Abstract

**Background:**

Risk prediction rules are important to establish appropriate treatment and management strategy for patients with different risk classification of pulmonary embolism (PE). Neutrophils are considered to be related to PE as an essential marker of inflammation. However, few studies have reported the association between neutrophil levels and risk classification of acute PE (APE). The aim of this study was to investigate the role of neutrophil levels upon admission in the assessment of risk classification of APE.

**Methods:**

A total of 299 consecutive APE patients and 90 patients without APE confirmed by computed tomographic pulmonary angiography were retrospectively screened. APE patients were stratified into two subgroups according to clinical guidelines: low- (*n* = 233) and intermediate- and high-risk (*n* = 60) APE.

**Results:**

The neutrophil levels in intermediate- and high-risk APE patients were significantly higher compared to low-risk APE or non-APE patients (*P* < 0.001). In multivariable logistic regression analysis, neutrophil levels were significantly and independently associated with intermediate- and high-risk APE (odds ratio = 1.239, 95% confidence interval [CI] 1.055–1.455, *P* = 0.009). Neutrophil levels were positively correlated with the pulmonary embolism severity index score (*r* = 0.357, *P* < 0.001), high sensitive C-reactive protein, D-dimer and pulmonary artery obstruction index (PAOI), in the overall population of APE patients. Receiver-operating characteristic curve analysis revealed that neutrophils had a better diagnostic value for intermediate- and high-risk APE (area under the curve [AUC] = 0.760, 95% CI 0.695–0.826; *P* < 0.001) compared to PAOI (AUC = 0.719) and D-dimer (AUC = 0.645).

**Conclusions:**

High neutrophil levels upon admission were significantly and independently associated with intermediate- and high-risk APE, which could be regarded as an indicator of inflammation and thrombosis in APE simultaneously. The potent diagnostic role of neutrophil levels and their competitive advantage over PAOI and D-dimer for the assessment of APE risk classification are suggested.

**Supplementary Information:**

The online version contains supplementary material available at 10.1186/s12959-023-00471-w.

## Introduction

Pulmonary embolism (PE) is one of the manifestations of venous thromboembolism (VTE) together with deep vein thrombosis (DVT) [1]. PE is considered to be the third most common cause of death from cardiopulmonary disease after acute myocardial infarction and stroke and has a mortality rate of 15–20% [2]. Patients in acute stages of PE are more at risk with a 30-day mortality rate of > 15% [3]. Although the 30-day mortality rate is currently decreasing [4, 5], nearly 94% of those who die due to APE sucumb prior to diagnosis of APE resulted from poor specificity of signs and symptoms [6, 7]. Furthermore, PE risk stratification, which refers to the severity of the actual APE, according to the ESC guidelines [8], can indicate the risk of early (in-hospital or 30-day) death in patients. Risk classification of PE is important to establish appropriate treatment and management strategy for patients [8]. Patients at intermediate or high risk may receive close surveillance in intensive care settings, including reperfusion treatment and haemodynamic support, whereas low-risk patients may be discharged earlier and receive home treatment [9]. Therefore, risk prediction rules for PE should be explored further.

In recent years, PE has been found to be accompanied by inflammation. Neutrophils are an essential indicator of inflammation that has been reported to be associated with PE. Elevated neutrophil and neutrophil–lymphocyte ratio (NLR) levels have been observed in patients with PE [10, 11]. Our previous study found that the percentage and levels of neutrophils, as well as markers of neutrophil extracellular traps (NETs), were significantly increased in patients with acute pulmonary embolism (APE) [12]. Furthermore, the prognostic role of NLR was also investigated in patients presenting with APE. NLR was shown to be an independent predictor of early mortality [13–15]. Nevertheless, unlike NLR, which remained stable both short and long term, neutrophils, which presented absolute neutrophil count, were not stable over time and were correlated with mortality in APE [13–15], reflecting the current inflammatory reaction. The association between neutrophil levels at the time of admission and risk classification of APE remains unknown. In addition, neutrophils and NETs have been reported to play an essential role in thrombosis [16, 17]. The pulmonary artery obstruction index (PAOI) obtained from CT pulmonary angiography (CTPA) images indicated the thrombus burden in APE patients. D-dimer was regarded as an indicator of the level of secondary fibrinolysis and the presence of venous thrombus. However, in the guidelines, D-dimer levels are not included in the biomarkers useful for the risk classification assessment. And the ability of PAOI and D-dimer to provide information for the assessment of APE risk classification remains controversial. The association between neutrophils and these parameters related to thrombosis should also be investigated.

We hypothesized that neutrophil levels strongly correlate with PE risk classification. Therefore, the present study sought to investigate the ability of neutrophil levels upon admission in determining the risk stratification of APE in comparison to other inflammatory and thrombotic markers of APE.

## Materials and methods

### Study population

We retrospectively screened 455 consecutive patients who were admitted to Fuwai Hospital (National Center for Cardiovascular Diseases, Beijing, China) and underwent CTPA on admission prior to any therapy measures between October 2014 and November 2019. A total of 299 patients diagnosed with APE and 90 patients without APE were included in this study. The exclusion criteria were as follows: age of < 18 years; no available laboratory or clinical data; active infection; acute myocardial infarction; autoimmune diseases; inflammatory rheumatic disease; hematological diseases; severe renal or liver disease; other systemic inflammatory diseases; and trauma or recent surgery. Patients who received immune suppressant treatment, had a recent blood transfusion, or used anti-inflammatory drugs were also excluded in consideration of the potential effect on the level of total and differential leukocyte counts. The diagnostic criteria and risk stratification standard for APE were in accordance with the European Society of Cardiology (ESC) guidelines [8, 18]. This study complied with the Declaration of Helsinki and the Ethics Committee of the hospital approving the protocol. All participants provided written informed consent before enrollment.

### Imaging data

CTPA was conducted for every subject to determine PE burden (saddle, main, lobar, segmental, and subsegmental). Scans were carried out using a 64-row spiral CT (Light Speed VCT, GE Healthcare, Milwaukee, WI, USA). A total of 100 mL of contrast medium (Omnipaque-300) was injected into the elbow vein at a flow rate of 4 mL/s. Lungs were scanned from the base to the apex in the caudocephalic direction with the tube voltage of 120 kV during an inspiratory breath hold. The presence of thrombus and the thrombus burden were interpreted by two experienced radiologists specifically trained in thoracic imaging who were blinded to the echocardiographic and biochemical results. Imaging data were evaluated according to the Fleischner Society’s Glossary of Terms [19]. The presence of pulmonary embolism showed a partial intraluminal defect surrounded by contrast medium or complete occlusion of the pulmonary artery in two consecutive CT sections. Thrombotic obstruction of the pulmonary arteries was calculated as PAOI according to the Qanadli et al. score [20].

### Biochemical and clinical measurements

Venous blood samples were drawn by strictly following a standard procedure at baseline before CTPA and prior to any therapy measures and then sent to the Laboratory Medicine Center of Fuwai Hospital for immediate testing. Routine blood tests including total and differential leukocyte counts, platelet (PLT) and hemoglobin (HGB) levels, and red cell distribution width (RDW) were performed using Sysmex XN 2000 automated hematology analyzer (Sysmex, Kobe, Japan). Serum total cholesterol (TC), triglycerides (TG), low-density lipoprotein cholesterol (LDL-C), high-sensitivity C-reactive protein (hsCRP), creatinine (CREA), aspartate transaminase (AST), and alanine transaminase (ALT) were assayed using Olympus AU-5400 biochemistry auto-analyzer (Olympus Corporation, Mishama, Japan). Plasma NT-proBNP was measured using a dedicated kit (NT-proBNP assays; Biomedica, Vienna, Austria). D-dimer was detected using STA R Max automated coagulation analyzer (Diagnostica Stago, Chausson, France). The NLR and PLR values were calculated according to neutrophil, platelet, and lymphocyte counts. The left ventricular ejection fraction (LVEF) and right ventricular diameter were determined according to the biplane Simpson rule via echocardiography.

### Statistical analysis

Statistical data analysis was performed using SPSS version 21.0 (SPSS Inc., Chicago, IL, USA). The results were presented as mean ± standard deviation (SD) for continuous variables if the data were normally distributed or as median and interquartile range (IQR, percentile_25-75_) if the data had a skewed distribution. All data were analyzed using independent-samples t-test, one-way ANOVA, or the Mann–Whitney U-test, as appropriate. Categorical variables were expressed as number, n (proportion, %) and assessed by chi-square or Fisher’s exact tests. Bivariate correlation analysis was performed with Pearson or Spearman’s correlation analysis. Multivariate logistic regression analysis was performed to identify the independent association between the risk factors and intermediate- and high-risk APE. Receiver-operating characteristic (ROC) curves were used to evaluate the predictive value of variables for APE patients with intermediate and high risk. Youden’s index was calculated as sensitivity + specificity-1 to determine the optimal cut-off value. A double-sided *P*-value of < 0.05 was considered to be statistically significant.

## Results

### Baseline characteristics

The study population included 389 patients undergoing CTPA (average age: 65.1 ± 12.5 years; range: 19–95 years; 38.8% males). Among the 389 patients, 299 patients were diagnosed with APE and 90 patients were included in the non-APE group confirmed by CTPA. APE patients were divided into low-risk (*n* = 233) and intermediate- and high-risk (*n* = 66) groups according to the ESC guidelines[8]. The baseline participant characteristics are summarized in Table 1. There were no significant differences among the three groups with respect to sex, LVEF, and hypertension frequency. Patients in the low-risk APE group were the oldest (*P* = 0.010). The prevalence of diabetes, hyperlipidemia, and coronary artery disease in the APE group was significantly lower than that in the non-APE group. However, there were no significant differences in the prevalence of the three diseases mentioned above between the two risk class APE groups. In the patients with intermediate- and high-risk APE, alcoholic drinkers accounted for a greater proportion than in low-risk APE patients (13.6% vs. 6.0%, *P* = 0.040). Higher heart beat levels, right ventricular diameter (RV), RV/LV, and PAOI (*P* < 0.001) and lower levels of systolic blood pressure (SBP; 115.8 ± 21.4 vs. 131.7 ± 22.9, *P* < 0.001) were also observed. When analyzing medications, the use of statin, aspirin, angiotensin II receptor blockers/angiotensin-converting enzyme inhibitors (ARB/ACEI), β-blocker, calcium antagonists, and diuretics did not exhibit significant differences between the two APE groups. However, non-APE patients used these drugs (except diuretics) more frequently compared to APE patients.

**Table 1:** Baseline study population characteristics

Variables	Non-APE group (*n* = 90)	Low-risk APE group (*n* = 233)	Intermediate- and high-risk APE group (*n* = 66)	*P*
Demographic				
Age, yrs	62.9 ± 11.5	66.7 ± 12.2	62.7 ± 13.8	0.010
Male, n (%)	41(45.6)	80(34.3)	30(45.5)	0.086
Smoking, n (%)	33(36.7)	33(14.2)	13(19.7)	< 0.001
Drinking, n (%)	28(31.1)	14(6.0)	9(13.6)	< 0.001
Clinical				
Hypertension, n (%)	61(67.8)	143(61.4)	37(56.1)	0.317
Diabetes, n (%)	25(27.8)	35(15.0)	11(16.9)	0.027
Hyperlipidemia, n (%)	60(66.7)	116(49.8)	37(56.1)	0.023
CAD, n (%)	62(68.9)	74(31.8)	18(27.3)	< 0.001
SBP, mmHg	131.8 ± 20.1	131.7 ± 22.9	115.8 ± 21.4	< 0.001
Heart rate, beats/min	71.4.0 ± 13.2	81.7 ± 17.7	95.2 ± 20.2	< 0.001
Right ventricular diameter, mm	22.5 ± 4.0	25.6 ± 6.0	28.0 ± 5.5	< 0.001
LVEF, %	61.3 ± 9.2	60.6 ± 8.5	59.7 ± 7.8	0.514
RV/LV	0.45(0.42, 0.50)	0.51(0.46, 0.66)	0.63(0.53, 0.75)	< 0.001
PAOI (%)	0(0, 0)	47.5(25.0, 67.5)	70.0(60.0, 75.0)	< 0.001
Laboratory				
TC, mmol/L	4.0 ± 1.0	4.3 ± 1.0	4.4 ± 1.1	0.018
TG, mmol/L	1.4(1.1, 2.0)	1.4(1.1, 1.8)	1.4(1.1, 2.0)	0.471
LDL-C, mmol/L	2.3(1.8, 2.9)	2.7(2.2, 3.2)	2.8(2.2, 3.4)	< 0.001
HsCRP, mg/L	2.1(1.1, 3.6)	6.2(3.0, 10.9)	11.1(5.6, 12.8)	< 0.001
Creatinine, µmol/L	79.1 ± 24.0	79.4 ± 20.6	80.6 ± 19.4	0.904
AST, U/L	20.0(16.0, 25.0)	23.0(18.0, 32.0)	22.0(16.0, 36.2)	0.005
ALT, U/L	21.0(14.0, 31.0)	21.0(13.0, 37.0)	28.0(17.8, 43.2)	0.052
WBC, 10^9^ cells/L	5.9(5.2, 7.6)	7.6(6.3, 9.2)	10.0(8.4, 11.9)	< 0.001
Percentage of neutrophils, %	61.0 ± 7.3	68.4 ± 10.1	73.1 ± 9.3	< 0.001
Neutrophils, 10^9^ cells/L	3.7(2.9, 4.8)	5.1(3.9, 6.6)	7.2(6.1, 9.1)	< 0.001
Percentage of lymphocytes, %	28.5(25.5, 33.3)	22.2(16.9, 29.1)	17.8(13.8, 23.4)	< 0.001
Lymphocytes, 10^9^ cells/L	1.8(1.5, 2.2)	1.6(1.3, 2.1)	1.9(1.4, 2.2)	0.090
Monocytes, 10^9^ cells/L	0.4(0.3, 0.5)	0.4(0.3, 0.6)	0.5(0.4, 0.7)	< 0.001
PLT, 10^9^/L	208.0(174.8, 258.5)	206.0(158.5, 243.0)	201.0(168.5, 256.8)	0.617
NLR	2.1(1.7, 2.6)	3.1(2.2, 4.5)	4.2(2.7, 5.8)	< 0.001
PLR	118.1(92.4, 146.3)	119.4(90.2, 166.1)	124.2(90.2, 143.3)	0.692
HGB, g/L	137.3 ± 18.6	134.5 ± 17.8	138.2 ± 19.3	0.236
RDW, %	13.0(12.3, 13.4)	13.1(12.4, 14.0)	13.0(12.5, 13.9)	0.115
D-dimer, ng/mL	0.9(0.5, 1.2)	3.1(1.7, 6.1)	5.9(2.7, 8.6)	< 0.001
NT-proBNP, pg/mL	139.0(88.9, 294.9)	533.8(144.7, 1268.05)	2093.9(787.8, 3386.3)	< 0.001
Medications				
Statins, n (%)	60(66.7)	49(21.0)	15(22.7)	< 0.001
Aspirin, n (%)	53(58.9)	34(14.6)	9(13.6)	< 0.001
ARB/ACEI, n (%)	42(46.7)	57(24.5)	12(18.2)	< 0.001
β-blocker, n (%)	52(57.8)	49(21.0)	13(19.7)	< 0.001
Calcium Antagonists, n (%)	36(40.0)	45(19.3)	15(22.7)	0.001
Diuretics, n (%)	16(19.3)	21(9.0)	7(10.6)	0.082

Values are the mean ± SD if the distribution is normal, and median (interquartile range) if skewed; number, n (proportions, %) for categorical variables

*ACEI* Angiotensin-converting enzyme inhibitor; *ALT* Alanine transaminase, *APE* Acute pulmonary embolism, *AST* Aspartate transaminase, *ARB* Angiotensin receptor blocker, *CAD* Coronary artery disease, *HGB* Hemoglobin, *HsCRP* High sensitive C-reactive protein, *LDL-C* Low-density lipoprotein cholesterol, *LV* Left ventricular, *LVEF* Left ventricular ejection fraction, *NLR* Neutrophil–lymphocyte ratio, *NT-proBNP* N-terminal pro-brain natriuretic peptide, *PAOI* Pulmonary artery obstruction index, *PLT* Platelet, *PLR* Platelet-lymphocyte ratio, *RDW* Red blood cell distribution width, *RV* Right ventricular, *SBP* Systolic blood pressure, *TC* Total cholesterol, *TG* Triglycerides, *WBC* White blood cell

**Fig. 1:**
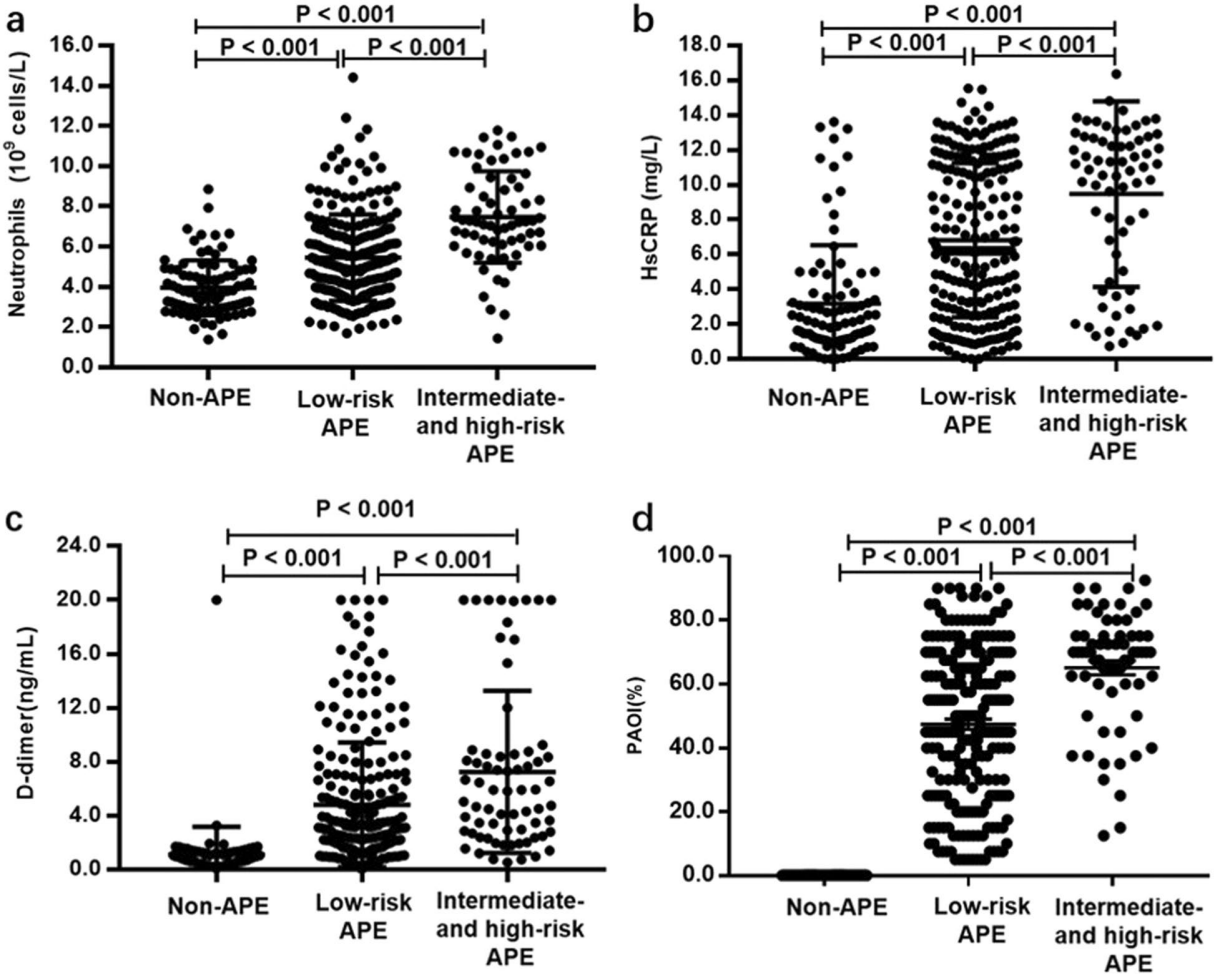
Levels of inflammatory and thrombotic markers according to clinical diagnosis and risk stratification of APE. **a** neutrophils; (**b**) hsCRP; (**c**) D-dimer; (**d**) PAOI. APE: acute pulmonary embolism; hsCRP: high sensitivity C-reactive protein. PAOI: pulmonary artery obstruction index. The inflammatory markers, including neutrophils and hsCRP, and thrombotic markers including D-dimer and PAOI, showed significant increases in intermediate- and high-risk APE group

According to the biochemical and hematological analysis results, significant increases in TC, LDL-C, NT-proBNP, and D-dimer levels were found in the APE group, especially in the intermediate- and high-risk APE patients. The inflammatory biomarkers, including white blood cells (WBC), neutrophils, monocytes, NLR, and hsCRP, were notably increased in patients with intermediate- and high-risk APE compared to low-risk APE or non-APE patients (*P* < 0.001). The levels of neutrophils and other inflammatory and thrombotic markers according to clinical diagnosis and risk stratification are shown in Fig. 1.

### Association between neutrophil levels and APE risk classification

**Fig. 2:**
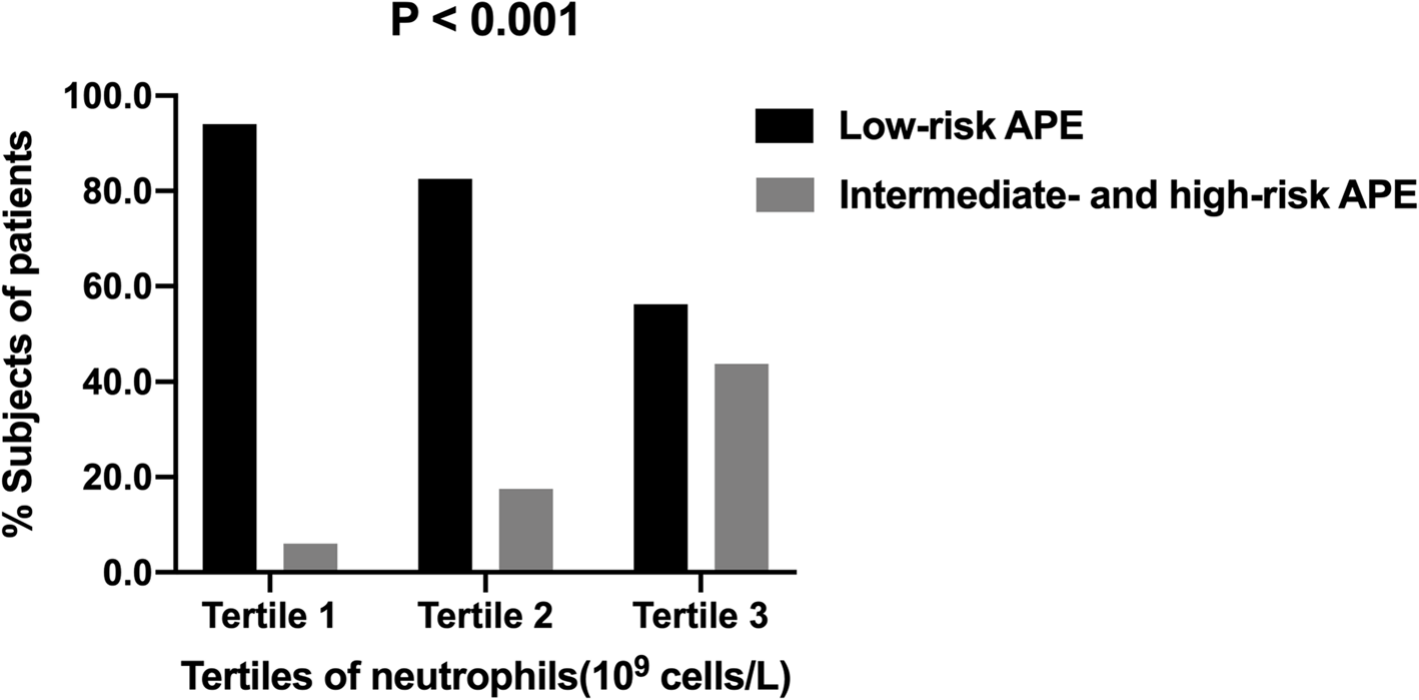
Percentage of low-risk APE patients and intermediate- and high-risk APE patients according to neutrophil tertiles. Percentages of patients with low-risk APE were higher in tertile 1 and 2 groups. Percentage of patients with intermediate- and high-risk APE gradually increased from 6.0% in tertile 1 to 43.8% in tertile 3

In view of the predominant increase in neutrophil levels in the APE group, especially in the intermediate- and high-risk compared to low-risk APE group, the role of neutrophils in indicating APE risk classification became a noteworthy focus of the study. To investigate the association between neutrophils and APE risk classification further, APE patients were stratified into three subgroups according to neutrophil tertiles. Demographic and clinical characteristics of APE patients according to neutrophil tertiles are reported in Table 2. The mean age and percentage of males in the study population did not differ significantly across the three subgroups. It is evident that the levels of PAOI, heart rate, hsCRP, D-dimer, and NT-proBNP were significantly different with ascending neutrophil levels (*P* < 0.001). Furthermore, percentages of low-risk and intermediate- and high-risk APE patients in the whole APE cohort were calculated. As shown in Fig. 2, the percentage of patients with intermediate- and high-risk APE gradually increased from 6.0% in tertile 1 to 43.8% in tertile 3 (*P* < 0.001). Patients with low-risk APE were more likely to be in tertile 1 and 2 groups (*P* < 0.001).

**Table 2:** Clinical and demographic characteristics of APE patients according to neutrophil tertiles

Variables	Tertile 1 (≤ 4.7) (*n* = 100)	Tertile 2 (> 4.7 and ≤ 6.7) (*n* = 103)	Tertile 3 (> 6.7) (*n* = 96)	*P*
Age, yrs	66.2 ± 11.8	66.0 ± 13.9	65.2 ± 12.3	0.877
Male, n (%)	36(36.0)	38(36.9)	36(37.5)	0.976
PAOI (%)	35.0(18.1, 45)	55.0(40.0, 70.0)	70.0(62.5, 80.0)	< 0.001
RV/LV	0.51(0.45, 0.65)	0.54(0.46, 0.71)	0.59(0.48, 0.71)	0.095
SBP, mmHg	129.8 ± 22.5	140.0 ± 24.2	123.6 ± 23.3	0.063
Heart rate, beats/min	75.2 ± 15.2	84.9 ± 17.4	94.4 ± 19.5	< 0.001
HsCRP, mg/L	4.5(1.7, 8.4)	6.2(3.2, 11.1)	10.9(6.2, 12.5)	< 0.001
Creatinine, µmol/L	78.0 ± 20.2	80.2 ± 20.0	80.8 ± 20.9	0.599
D-dimer, ng/mL	2.4(1.4, 3.6)	3.2(2.1, 7.1)	5.9(3.0, 10.8)	< 0.001
NT-proBNP, pg/mL	533.8(117.2, 1075.8)	533.8(182.0, 2105.6)	1108.3(533.8, 3106.0)	< 0.001
Low-risk APE, n (%)	94(94.0)	85(82.5)	54(56.2)	< 0.001
Intermediate- and High-risk APE, n (%)	6(6.0)	18(17.5)	42(43.8)	< 0.001

Abbreviations as in Table 1

Given the differences in neutrophil levels based on the APE risk stratification, three regression models were subsequently established to determine if the neutrophil level upon admission is a significant predictor for intermediate- and high-risk APE (Table 3). As a result of univariate regression analysis of model 1 in all individuals with APE, PAOI, hsCRP, D-dimer, neutrophils, and their tertiles were found to be statistically significant predictors. They were also independently associated with intermediate- and high-risk APE after adjustment for age, drinking, SBP, heart rate, RV/LV, and NT-proBNP in model 2. Furthermore, in a fully adjusted multivariate regression of model 3, PAOI, neutrophils, and their tertile 3 were significantly independent with OR values of 1.018 (95% CI: 1.000–1.036, *P* = 0.044), 1.239 (95% CI: 1.055–1.455, *P* = 0.009), and 5.440 (95% CI: 1.748–16.993, *P* = 0.003), respectively, whereas hsCRP and D-dimer were not significantly independent.

**Table 3:** Regression analysis of clinical and hematologic parameters for prediction of intermediate- and high-risk APE^a^

Parameters	Model 1		Model 2		Model 3	
	OR (95% CI)	*P*	OR (95% CI)	*P*	OR (95% CI)	*P*
PAOI	1.039(1.024–1.055)	< 0.001	1.030(1.014–1.047)	< 0.001	1.018(1.000–1.036)	0.044
D-dimer	1.091(1.038–1.146)	0.001	1.066(1.008–1.127)	0.025	1.016(0.956–1.080)	0.613
HsCRP	1.136(1.067–1.210)	< 0.001	1.098(1.028–1.172)	0.006	1.045(0.973–1.123)	0.226
Neutrophil count	1.457(1.281–1.658)	0.024	1.363(1.179–1.577)	< 0.001	1.239(1.055–1.455)	0.009
Neutrophil count tertiles						
Tertile 1	1		1		1	
Tertile 2	3.318(1.258–8.746)	0.015	3.154(1.128–8.824)	0.029	2.377(0.802–7.046)	0.118
Tertile 3	12.185(4.863–30.531)	< 0.001	9.335(3.429–25.417)	< 0.001	5.440(1.748–16.933)	0.003

*CI* Confidence interval, *OR* Odds ratio; other abbreviations as in Table 1

^a^Model 1: Unadjusted; Model 2: Adjusted for age, drinking, SBP, heart rate, RV/LV, and NT-proBNP; Model 3: Adjusted for age, drinking, SBP, heart rate, RV/LV, NT-proBNP, and mutually for the other three parameters

### Correlation between neutrophil levels and parameters associated with APE risk classification

Pulmonary embolism severity index (PESI) score, RV/LV and NT-proBNP have been recommended to be associated with APE risk classification. The correlation analysis of clinical and hematologic parameters including neutrophils, with those parameters associated with APE risk classification, was performed. Neutrophil levels were positively correlated with the PESI score (*r* = 0.357, *P* < 0.001), yet weakly correlated with RV/LV (*r* = 0.151, *P* = 0.009), in the overall population of APE patients (Table 4). Furthermore, significant and positive correlations were found between neutrophils and inflammatory marker, hsCRP (*r* = 0.420, *P* < 0.001), and thrombotic markers including D-dimer (*r* = 0.370, *P* < 0.001) and PAOI (*r* = 0.566, *P* < 0.001). It was also positively correlated with NT-proBNP (*r* = 0.289, *P* < 0.001), which has been suggested to be a laboratory APE biomarker in the guidelines. Despite that, hsCRP, D-dimer, PAOI and NT-proBNP were also significantly correlated with the PESI score, while neutrophil levels had the strongest correlation with the PESI score. Notably, there was a relatively stronger correlation between NT-proBNP and RV/LV (*r* = 0.356, *P* < 0.001) compared to the other clinical and hematologic biomarkers.

**Table 4:** Correlation analysis of clinical and hematologic parameters with PESI score, RV/LV and NT-proBNP in APE patients

Variables		PESI score	RV/LV	NT-proBNP	Neutrophil count	HsCRP	D-dimer	PAOI
PESI score	Spearman correlation	1	0.272	0.354	0.357	0.252	0.270	0.336
	P		<0.001	<0.001	<0.001	<0.001	<0.001	<0.001
RV/LV	Spearman correlation	0.272	1	0.356	0.151	0.158	0.164	0.341
	P	<0.001		<0.001	0.009	0.006	0.004	<0.001
NT-proBNP	Spearman correlation	0.354	0.356	1	0.289	0.293	0.266	0.346
	P	<0.001	<0.001		<0.001	<0.001	<0.001	<0.001
Neutrophil count	Spearman correlation	0.357	0.151	0.289	1	0.420	0.370	0.566
	P	<0.001	0.009	<0.001		<0.001	<0.001	<0.001
HsCRP	Spearman correlation	0.252	0.158	0.293	0.420	1	0.342	0.340
	P	<0.001	0.006	<0.001	<0.001		<0.001	<0.001
D-dimer	Spearman correlation	0.270	0.164	0.266	0.370	0.342	1	0.440
	P	<0.001	0.004	<0.001	<0.001	<0.001		<0.001
PAOI	Spearman correlation	0.336	0.341	0.346	0.566	0.340	0.440	1
	P	<0.001	<0.001	<0.001	<0.001	<0.001	<0.001	

*Correlation is significant at the 0.05 level of *P*-value

*PESI* pulmonary embolism severity index. Other abbreviations as in Table 1

### Diagnostic value of admission neutrophil levels for intermediate- and high-risk APE

**Fig. 3:**
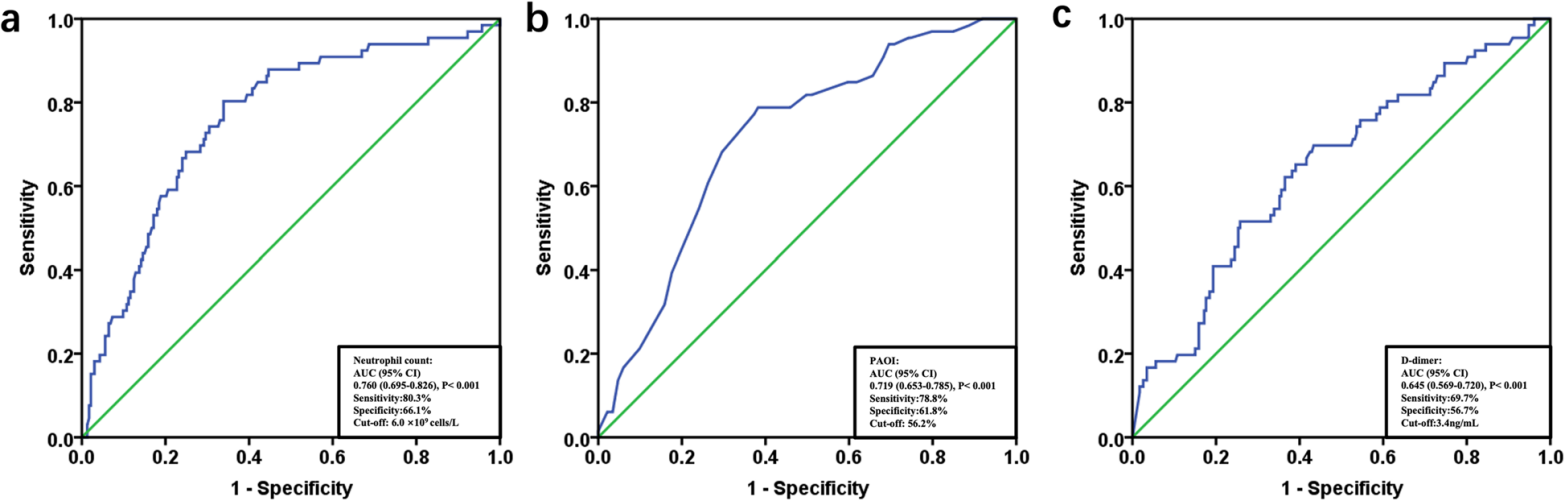
Receiver operating characteristic (ROC) curve for admission neutrophil level (**a**), PAOI (**b**), and D-dimer (**c**) for intermediate- and high-risk APE in the whole APE patient cohort. Admission neutrophil level had the best diagnostic value for intermediate- and high-risk APE in the overall APE population

ROC curve analysis was performed to evaluate the diagnostic value of neutrophil level for intermediate- and high-risk APE (Fig. 3). The diagnostic values of PAOI and D-dimer were also analyzed. The results revealed that the admission neutrophil level had the best diagnostic value for intermediate- and high-risk APE in the overall APE population with a sensitivity of 80.3% and a specificity of 66.1% (AUC = 0.760, 95% CI 0.695–0.826; *P* < 0.001). The optimal cut-off value for the admission neutrophil level to distinguish intermediate- and high-risk from low-risk APE was 6.0 × 10^9^ cells/L. The AUC values for PAOI and D-dimer for intermediate- and high-risk APE were 0.719 (95% CI 0.653–0.785; *P* < 0.001) and 0.645 (95% CI 0.569–0.720; *P* < 0.001), respectively.

## Discussion

Prior works have documented the role of neutrophils in inflammation [21–23] and the association between neutrophils and PE. The association between elevated WBC levels in PE patients was first described by Afzal et al. [24]. Moreover, previous experimental studies have demonstrated that NLR levels in APE patients were higher than those in healthy controls or patients without APE [10, 11]. However, these studies have not focused on the relationship between the neutrophils and APE risk classification or made a comparison with other parameters related to APE. To the best of our knowledge, this is the first study to investigate the role of neutrophils in the assessment of APE risk classification and investigate the association between neutrophils and inflammation and thrombosis in APE simultaneously.

The present study clarified that the admission neutrophil levels were significantly higher in APE patients than in non-APE patients, which was confirmed by CTPA. In recent years, the function of inflammation in VTE in addition to acute coronary syndrome has become a topic of investigation [25]. The present observation of significant elevation in neutrophil levels and hsCRP in patients provided evidence for the role of inflammation in APE. This is in accordance with the JUPITER Trial [26]. Interestingly, patients on statin drugs were significantly less likely to have a PE in this study of seemingly healthy patients and had lower LDL, which is also in keeping with the JUPITER Trial [26]. In addition, a significantly positive correlation between neutrophil and hsCRP levels implied that neutrophils can also reflect the extent of the inflammatory response in the host.

Importantly, neutrophil levels in the present study exhibited an ascending trend in intermediate- and high-risk APE after risk stratification. In addition, a promising diagnostic performance of neutrophils was identified with increasing AUC, sensitivity, and specificity compared to PAOI and D-dimer. The markedly positive correlation between neutrophils and PAOI or D-dimer levels suggested a potential role for neutrophil levels in venous thrombogenic activity. D-dimer testing, which reflects the level of secondary fibrinolysis and detects the presence of venous thrombus, was used extensively in conjunction with clinical decision rules [27, 28]. PAOI calculated from CTPA imaging reflected the APE thrombus burden [29]. Neutrophils have also been reported to participate in the process of thrombosis [10, 30]. Animal experiments have shown that neutrophils contribute to the pathogenesis of venous thrombosis. It has also been observed that endothelial dysfunction detected by brachial artery flow-mediated dilation is present in patients with pulmonary thromboembolism [10]. Activated leukocytes produce oxygen radicals and trigger endothelial injury, which increases inflammation and thrombosis in pulmonary embolism [31]. It has been reported that NLR is associated with massive embolism in patients with PE [15], indicating that it can be related to thrombotic burden of PE. Furthermore, activated neutrophils release neutrophil extracellular traps (NETs), inducing propagation of thrombosis [32]. Maurits L. van Montfoort et al. [33] identified an association among circulating nucleosomes, activated neutrophils, and the presence of DVT. Thus, neutrophils might also provide incremental information regarding the thrombotic degree in the progression of APE. It can therefore be implied that the positive correlation between neutrophils and D-dimer or PAOI is reasonable and evident.

In addition to laboratory biomarkers and imaging indicators, a positive correlation between neutrophil levels and PESI score, a clinical parameter, was also observed. It can thus be implied that the neutrophil level simultaneously reflects the inflammation and thrombosis levels, thus presenting a promising diagnostic parameter for intermediate- and high-risk APE. In contrast, D-dimer and PAOI only indicate thrombosis, which lack information regarding the level of inflammation. D-dimer testing has a high negative predictive value in which a normal D-dimer level renders acute PE or DVT unlikely. However, D-dimer risk stratification ability is low, which was also observed in the present study. Accordingly, the association between PAOI and APE risk classification remains controversial. The present study adopted the Qanadli score to calculate the value of PAOI, concluding that PAOI is associated with APE risk classification, which is consistent with Mahmoud M. Higazi1 et al. results [34]. In contrast, Marianne Lerche et al. used the Mastora score as PAOI [35] and have reported no correlation between PAOI and APE risk classification. Therefore, different PAOI scoring methods might impact the final results. Moreover, a sophisticated PAOI scoring method that requires an experienced radiologist and time limits the application of PAOI in the clinic.

### Clinical perspective

Neutrophil level testing can be regarded as a cheap and widely available indicator of inflammation and thrombosis. The present study provides diagnostic implications for intermediate- and high-risk APE in order to better understand the function role of neutrophils in APE. This introduces a new insight into the clinical diagnosis of APE risk classification and addresses the important role of neutrophil level changes in the progression of APE. The internal mechanisms of elevated neutrophil levels and inflammatory response in APE patients need to be further explored. Overall, the presence of this association should not be ignored by the clinicians during objective APE risk stratification and therapy management for APE patients with different risk stratification. Furthermore, many times patients who come in with hemodynamic shock (or who develop it in the ICU) and are suspected of having a massive PE are not stable enough to go to the CT scanner for a CT angiogram. The clinician is then faced with making a risk: benefit decision of using systemic lytic therapy with tPA based on clinical suspicion for massive PE. Based on our study, it would be worth attempting to generate a predictive calculator for likelihood of a PE being present that accounts for neutrophil levels to improve clinical diagnosis in the absence of CT scans, which would be explored in the future study.

### Study limitations

This study has several limitations. First, this was a cross-sectional single-center study, which cannot provide prognostic information. Second, because neutrophil levels could be affected by active infections, autoimmune/rheumatologic diseases, hematologic diseases, and other systemic inflammatory disorders. To avoid the effect on it, we excluded patients with these diseases. Therefore, the conclusion drawn by this study could be applicable to those patients without these diseases, which might be the limitation of applicable population. Given the lack of specificity of neutrophil levels, which are often elevated in response to any physiologic stress (including moderate and brief exercise), a repeat study similar to this but including all patient populations may render the finding non-useful. For the same reason, many of the groups of patients who would be high risk for developing a PE were excluded in the first place. Thus, intermediate- and high-risk patients were combined into one group. Further studies in a larger population and a more detailed grouping are needed to verify these findings, for example whether neutrophils can distinguish high-risk from intermediate-risk APE. Third, we used the upper limit of D-dimer to describe out of range values due to the limitation of clinical assay. The data of D-dimer had a skewed distribution and Mann–Whitney U-test was used to analyze the difference of D-dimer levels among groups. Therefore, there was no impact on this result. We have also made sensitivity analysis to evaluate the impact on the other statistical analysis, in which patients with D-dimer > 20 ng/mL were excluded. Some important results were shown in Supplementary materials. It could be found that there was no obvious impact on P values, so the impact of D-dimer could be ignored. In addition, the study findings cannot be extrapolated to other ethnic groups. Therefore, the association between elevated neutrophil level and APE risk classification in other populations should be studied in the future.

## Conclusions

In summary, a high neutrophil level upon admission is significantly and independently associated with intermediate- and high-risk APE, which could be regarded as an indicator of inflammation and thrombosis in APE simultaneously. The potent diagnostic role of neutrophil level upon admission and its competitive advantage over PAOI and D-dimer for the assessment of APE risk classification, suggesting that it could be applied in clinics, including as part of a decision making pathway with regards to who gets a CTPA study or not and for QI analysis.

## Supplementary Information

**Additional file1:Table S1.** Regression analysis of clinical and hematologic parameters for prediction of intermediate- and high-risk APE^a^. **Table S2.** Correlation analysis of D-dimer with PESI score, RV/LV and NT-proBNP in APE patients. **Table S3.** Receiver operating characteristic (ROC) curve data for admission D-dimer and neutrophils for intermediate- and high-risk APE in the whole APE patient cohort.

## Data Availability

The datasets generated and/or analyzed during the current study are not publicly available due to subsequent researchers based on this data base not being published but are available from the corresponding author on reasonable request.

## Abbreviations

ACEI: Angiotensin-converting enzyme inhibitor
ALT: Alanine transaminase
APE: Acute pulmonary embolism
ARB: Angiotensin receptor blocker
AST: Aspartate transaminase
AUC: Area under the curve
CAD: Coronary artery disease
CI: Confidence interval
CTPA: CT pulmonary angiography
DVT: Deep vein thrombosis
ESC: European Society of Cardiology
HGB: Hemoglobin
HsCRP: High sensitive C-reactive protein
LDL-C: Low-density lipoprotein cholesterol
LV: Left ventricular
LVEF: Left ventricular ejection fraction
NETs: Neutrophil extracellular traps
NLR: Neutrophil–lymphocyte ratio
NT-proBNP: N-terminal pro-brain natriuretic peptide
OR: Odds ratio
PAOI: Pulmonary artery obstruction index
PE: Pulmonary embolism
PLT: Platelet
PLR: Platelet-lymphocyte ratio
RDW: Red blood cell distribution width
ROC: Receiver-operating characteristic
RV: Right ventricular
SBP: Systolic blood pressure
TC: Total cholesterol
TG: Triglycerides
VTE: Venous thromboembolism
WBC: White blood cell

## References

[1] Di Nisio M, van Es N, Buller HR (2016). Deep vein thrombosis and pulmonary embolism. Lancet.

[2] Goldhaber SZ, Visani L, De Rosa M (1999). Acute pulmonary embolism: clinical outcomes in the International Cooperative Pulmonary Embolism Registry (ICOPER). Lancet.

[3] Torbicki A, Perrier A, Konstantinides S, Agnelli G, Galiè N, Pruszczyk P (2008). Guidelines on the diagnosis and management of acute pulmonary embolism: the Task Force for the Diagnosis and Management of Acute Pulmonary Embolism of the European Society of Cardiology (ESC). Eur Heart J.

[4] Søgaard KK, Schmidt M, Pedersen L, Horváth-Puhó E, Sørensen HT (2014). 30-year mortality after venous thromboembolism: a population-based cohort study. Circulation.

[5] Stein PD, Matta F, Alrifai A, Rahman A (2012). Trends in case fatality rate in pulmonary embolism according to stability and treatment. Thromb Res.

[6] Dalen JE (2002). Pulmonary embolism: what have we learned since Virchow? Natural history, pathophysiology, and diagnosis. Chest.

[7] Pollack CV, Schreiber D, Goldhaber SZ, Slattery D, Fanikos J, O'Neil BJ (2011). Clinical characteristics, management, and outcomes of patients diagnosed with acute pulmonary embolism in the emergency department: initial report of EMPEROR (Multicenter Emergency Medicine Pulmonary Embolism in the Real World Registry). J Am Coll Cardiol.

[8] Konstantinides SV, Meyer G, Becattini C, Bueno H, Geersing GJ, Harjola VP (2019). ESC Guidelines for the diagnosis and management of acute pulmonary embolism developed in collaboration with the European Respiratory Society (ERS): The Task Force for the diagnosis and management of acute pulmonary embolism of the European Society of Cardiology (ESC). Eur Respir J.

[9] Jaff MR, McMurtry MS, Archer SL, Cushman M, Goldenberg N, Goldhaber SZ (2011). Management of massive and submassive pulmonary embolism, iliofemoral deep vein thrombosis, and chronic thromboembolic pulmonary hypertension: a scientific statement from the American Heart Association. Circulation.

[10] Kurtipek E, Büyükterzi Z, Büyükterzi M, Alpaydın M, Erdem S (2017). Endothelial dysfunction in patients with pulmonary thromboembolism: neutrophil to lymphocyte ratio and platelet to lymphocyte ratio. Clin Respir J.

[11] Celik A, Ozcan I, Gündes A, Topuz M, Pektas I, Yesil E (2015). Usefulness of admission hematologic parameters as diagnostic tools in acute pulmonary embolism. Kaohsiung J Med Sci.

[12] Li T, Peng R, Wang F, Hua L, Liu S, Han Z (2020). Lysophosphatidic acid promotes thrombus stability by inducing rapid formation of neutrophil extracellular traps: A new mechanism of thrombosis. J Thromb Haemost.

[13] Ma Y, Mao Y, He X, Sun Y, Huang S, Qiu J (2016). The values of neutrophil to lymphocyte ratio and platelet to lymphocyte ratio in predicting 30 day mortality in patients with acute pulmonary embolism. BMC Cardiovasc Disord.

[14] Kayrak M, Erdogan HI, Solak Y, Akilli H, Gul EE, Yildirim O (2014). Prognostic value of neutrophil to lymphocyte ratio in patients with acute pulmonary embolism: a restrospective study. Heart Lung Circ.

[15] Soylu K, Gedikli O, Eksi A, Avcioglu Y, Soylu AI, Yuksel S (2016). Neutrophil-to-lymphocyte ratio for the assessment of hospital mortality in patients with acute pulmonary embolism. Arch Med Sci.

[16] Vazquez-Garza E, Jerjes-Sanchez C, Navarrete A, Joya-Harrison J, Rodriguez D (2017). Venous thromboembolism: thrombosis, inflammation, and immunothrombosis for clinicians. J Thromb Thrombolysis.

[17] Mangold A, Alias S, Scherz T, Hofbauer T, Jakowitsch J, Panzenböck A (2015). Coronary neutrophil extracellular trap burden and deoxyribonuclease activity in ST-elevation acute coronary syndrome are predictors of ST-segment resolution and infarct size. Circ Res.

[18] Konstantinides SV, Meyer G (2019). The 2019 ESC Guidelines on the Diagnosis and Management of Acute Pulmonary Embolism. Eur Heart J.

[19] Hansell DM, Bankier AA, MacMahon H, McLoud TC, Müller NL, Remy J (2008). Fleischner Society: glossary of terms for thoracic imaging. Radiology.

[20] Qanadli SD, El Hajjam M, Vieillard-Baron A, Joseph T, Mesurolle B, Oliva VL (2001). New CT index to quantify arterial obstruction in pulmonary embolism: comparison with angiographic index and echocardiography. AJR Am J Roentgenol.

[21] Angkananard T, Anothaisintawee T, Thakkinstian A (2017). Neutrophil lymphocyte ratio and risks of cardiovascular diseases: A systematic review and meta-analysis. Atherosclerosis.

[22] Wheeler JG, Mussolino ME, Gillum RF, Danesh J (2004). Associations between differential leucocyte count and incident coronary heart disease: 1764 incident cases from seven prospective studies of 30,374 individuals. Eur Heart J.

[23] Abakay O, Abakay A, Sen HS, Tanrikulu AC (2015). The relationship between inflammatory marker levels and pulmonary tuberculosis severity. Inflammation.

[24] Afzal A, Noor HA, Gill SA, Brawner C, Stein PD (1999). Leukocytosis in acute pulmonary embolism. Chest.

[25] Kunutsor SK, Seidu S, Blom AW, Khunti K, Laukkanen JA (2017). Serum C-reactive protein increases the risk of venous thromboembolism: a prospective study and meta-analysis of published prospective evidence. Eur J Epidemiol.

[26] Ridker PM, Danielson E, Fonseca FA, Genest J, Gotto AM, Kastelein JJ (2009). Reduction in C-reactive protein and LDL cholesterol and cardiovascular event rates after initiation of rosuvastatin: a prospective study of the JUPITER trial. Lancet.

[27] Wells PS, Anderson DR, Rodger M, Stiell I, Dreyer JF, Barnes D (2001). Excluding pulmonary embolism at the bedside without diagnostic imaging: management of patients with suspected pulmonary embolism presenting to the emergency department by using a simple clinical model and d-dimer. Ann Intern Med.

[28] Tang N, Sun Z, Li D, Yang J, Yin S, Guan Q (2017). Combined measurement of factor XIII and D-dimer is helpful for differential diagnosis in patients with suspected pulmonary embolism. Clin Chem Lab Med.

[29] Furlan A, Aghayev A, Chang CC, Patil A, Jeon KN, Park B (2012). Short-term mortality in acute pulmonary embolism: clot burden and signs of right heart dysfunction at CT pulmonary angiography. Radiology.

[30] Zarbock A, Polanowska-Grabowska RK, Ley K (2007). Platelet-neutrophil-interactions: linking hemostasis and inflammation. Blood Rev.

[31] Mühl D, Füredi R, Cristofari J, Ghosh S, Bogár L, Borsiczki B (2006). Evaluation of oxidative stress in the thrombolysis of pulmonary embolism. J Thromb Thrombolysis.

[32] Pfeiler S, Stark K, Massberg S, Engelmann B (2017). Propagation of thrombosis by neutrophils and extracellular nucleosome networks. Haematologica.

[33] van Montfoort ML, Stephan F, Lauw MN, Hutten BA, Van Mierlo GJ, Solati S (2013). Circulating nucleosomes and neutrophil activation as risk factors for deep vein thrombosis. Arterioscler Thromb Vasc Biol.

[34] Higazi MM, Fattah R, Abdelghany EA, Ghany HSA (2020). Efficacy of Computed Tomography Pulmonary Angiography as Non-invasive Imaging Biomarker for Risk Stratification of Acute Pulmonary Embolism. J Clin Imaging Sci.

[35] Lerche M, Bailis N, Akritidou M, Meyer HJ, Surov A (2019). Pulmonary Vessel Obstruction Does Not Correlate with Severity of Pulmonary Embolism. J Clin Med.

