# Supplementary material for: Neutrophil levels upon admission for the assessment of acute pulmonary embolism with intermediate- and high-risk: an indicator of thrombosis and inflammation
Source: Thromb J. 2023 Mar 14;21:28. doi: 10.1186/s12959-023-00471-w (PMC10015714; doi:10.1186/s12959-023-00471-w)
Supplement: Supplementary file 1 — Additional file1:Table S1. Regression analysis of clinical and hematologic parameters for prediction of intermediate- and high-risk APEa. Table S2. Correlation analysis of D-dimer with PESI score, RV/LV and NT-proBNP in APE patients. Table S3. Receiver operating characteristic (ROC) curve data for admission D-dimer and neutrophils for intermediate- and high-risk APE in the whole APE patient cohort. [file 12959_2023_471_MOESM1_ESM.docx]

**Supplementary material**

Table S1 Regression analysis of clinical and hematologic parameters for prediction of intermediate- and high-risk APE.^a^

| Parameters | Model 1 | |  | Model 2 | |  | Model 3 | |
| --- | --- | --- | --- | --- | --- | --- | --- | --- |
|  | **OR (95% CI)** | **P** |  | **OR (95% CI)** | **P** |  | **OR (95% CI)** | **P** |
| D-dimer | **1.091(1.038-1.146)** | **0.001** |  | **1.066(1.008-1.127)** | **0.025** |  | **1.016(0.956-1.080)** | **0.613** |
| D-dimer _a_ | **1.067(1.003-1.134)** | **0.040** |  | **1.032(0.962-1.107)** | **0.385** |  | **0.983(0.910-1.061)** | **0.654** |
| Neutrophil count | **1.457(1.281-1.658)** | **0.024** |  | **1.363(1.179-1.577)** | **<0.001** |  | **1.239(1.055-1.455)** | **0.009** |
| Neutrophil count _a_ | **1.492(1.301-1.711)** | **<0.001** |  | **1.408(1.206-1.643)** | **<0.001** |  | **1.421(1.211-1.667)** | **<0.001** |

CI: confidence interval; OR: odds ratio.

_a_: data in sensitivity analysis.

^a^ Model 1: Unadjusted; Model 2: Adjusted for age, drinking, SBP, heart rate, RV/LV, and NT-proBNP; Model 3: Adjusted for age, drinking, SBP, heart rate, RV/LV, NT-proBNP, and mutually for the other three parameters.

Table S2 Correlation analysis of D-dimer with PESI score, RV/LV and NT-proBNP in APE patients.

| **Variables** |  | **PESI score** | **RV/LV** | **NT-proBNP** | **Neutrophil count** | **HsCRP** | **D-dimer** | **PAOI** |
| --- | --- | --- | --- | --- | --- | --- | --- | --- |
| **D-dimer** | **Spearman correlation** | **0.270** | **0.164** | **0.266** | **0.370** | **0.342** | **1** | **0.440** |
|  | **P** | **<0.001** | **0.004** | **<0.001** | **<0.001** | **<0.001** |  | **<0.001** |
| **D-dimer_a_** | **Spearman correlation** | **0.233** | **0.167** | **0.242** | **0.352** | **0.303** | **1** | **0.425** |
|  | **P** | **<0.001** | **0.005** | **<0.001** | **<0.001** | **<0.001** |  | **<0.001** |
| **Neutrophil count** | **Spearman correlation** | **0.357** | **0.151** | **0.289** | **1** | **0.420** | **0.370** | **0.566** |
|  | **P** | **<0.001** | **0.009** | **<0.001** |  | **<0.001** | **<0.001** | **<0.001** |
| **Neutrophil count_a_** | **Spearman correlation** | **0.348** | **0.151** | **0.274** | **1** | **0.413** | **0.352** | **0.562** |
|  | **P** | **<0.001** | **0.010** | **<0.001** |  | **<0.001** | **<0.001** | **<0.001** |

_a_: data in sensitivity analysis.

* Correlation is significant at the 0.05 level of P-value.

Table S3 Receiver operating characteristic (ROC) curve data for admission D-dimer and neutrophils for intermediate- and high-risk APE in the whole APE patient cohort.

| **Variables** | **AUC(95%CI)** | **P** |
| --- | --- | --- |
| **D-dimer** | **0.645(0.569-0.720)** | **<0.001** |
| **D-dimer_a_** | **0.614(0.535-0.693)** | **0.007** |
| **Neutrophils** | **0.760(0.695-0.826)** | **<0.001** |
| **Neutrophils _a_** | **0.771(0.702-0.840)** | **<0.001** |
| **PAOI** | **0.719(0.653-0.785)** | **<0.001** |
| **PAOI_a_** | **0.716(0.647-0.785)** | **<0.001** |

_a_: data in sensitivity analysis.
